# Detection of *Burkholderia pseudomallei* O-antigen serotypes in near-neighbor species

**DOI:** 10.1186/1471-2180-12-250

**Published:** 2012-11-05

**Authors:** Joshua K Stone, Mark Mayo, Stephanie A Grasso, Jennifer L Ginther, Stephanie D Warrington, Christopher J Allender, Adina Doyle, Shalamar Georgia, Mirjam Kaestli, Stacey M Broomall, Mark A Karavis, Joseph M Insalaco, Kyle S Hubbard, Lauren A McNew, Henry S Gibbons, Bart J Currie, Paul Keim, Apichai Tuanyok

**Affiliations:** 1 Center for Microbial Genetics and Genomics, Northern Arizona University, Flagstaff, AZ, USA; 2 Menzies School of Health Research, Darwin, NT, Australia; 3 BioSciences Division, Edgewood Chemical Biological Center, Aberdeen Proving Ground, MD, USA

## Abstract

**Background:**

*Burkholderia pseudomallei* is the etiological agent of melioidosis and a CDC category B select agent with no available effective vaccine. Previous immunizations in mice have utilized the lipopolysaccharide (LPS) as a potential vaccine target because it is known as one of the most important antigenic epitopes in *B*. *pseudomallei*. Complicating this strategy are the four different *B. pseudomallei* LPS O-antigen types: A, B, B2, and rough. Sero-crossreactivity is common among O-antigens of *Burkholderia* species. Here, we identified the presence of multiple *B. pseudomallei* O-antigen types and sero-crossreactivity in its near-neighbor species.

**Results:**

PCR screening of O-antigen biosynthesis genes, phenotypic characterization using SDS-PAGE, and immunoblot analysis showed that majority of *B. mallei* and *B. thailandensis* strains contained the typical O-antigen type A. In contrast, most of *B. ubonensis* and *B. thailandensis*-like strains expressed the atypical O-antigen types B and B2, respectively. Most *B*. *oklahomensis* strains expressed a distinct and non-seroreactive O-antigen type, except strain E0147 which expressed O-antigen type A. O-antigen type B2 was also detected in *B*. *thailandensis* 82172, *B*. *ubonensis* MSMB108, and *Burkholderia* sp. MSMB175. Interestingly, *B*. *thailandensis*-like MSMB43 contained a novel serotype B positive O-antigen.

**Conclusions:**

This study expands the number of species which express *B. pseudomallei* O-antigen types. Further work is required to elucidate the full structures and how closely these are to the *B. pseudomallei* O-antigens, which will ultimately determine the efficacy of the near-neighbor B serotypes for vaccine development.

## Background

Lipopolysaccharide (LPS) is an amphiphilic molecule which is a major component in the outer membrane of Gram-negative bacteria [1]. It is composed of three parts – a membrane bound lipid A, or endotoxin, a core oligosaccharide, and a repeating O-antigen [2]. The lipid A is the signal that triggers the innate immune system during infection and is structurally conserved across genera with differences in immune response attributable to the presence of varying fatty acids [1,3,4]. The O-antigen is the most structurally diverse LPS component within a species, with over 170 known structures in *Escherichia coli* alone [1]. As an antigenic determinant, O-antigen structures can be grouped by serotype [2].

*Burkholderia pseudomallei* is a saprophytic Gram-negative bacterium endemic to Southeast Asia and Australia. It is the etiological agent of the septicemic disease melioidosis and a CDC category B select agent with no available effective vaccine [5,6]. However, limited success has been met with use of LPS from *B. pseudomallei* and the avirulent near-neighbor *B. thailandensis* in rodent and rabbit melioidosis models [7-10]. Four distinct O-antigen ladder patterns have been described in *B. pseudomallei*, known as types A, B, B2, and rough, which lacks the repeating unit [11]. Most *B. pseudomallei* strains express type A O-antigen, making it by far the most abundant structure, whereas the atypical types, B and B2, are serologically related but have distinct ladder banding patterns when run on SDS-PAGE [11]. Type A is also found in *B. thailandensis* and the virulent *B*. *mallei*[12,13]. This is also the only O-antigen that has been structurally characterized, containing a disaccharide 3)-β-D-glucopyranose-(1,3)-6d-α-L-talopyranose-(1 repeat, with the talose residue variably acetylated and methylated [13-16]. Type B has not been found in any other species while type B2 was recently described in a *B. thailandensis*-like species [11]. *B*. *thailandensis*-like species is a new species within the Pseudomallei phylogenetic group which is closely related to *B*. *pseudomallei* and *B. thailandensis.* This new species was first discovered in soil and water in northern Australia [17]. The presence of types A and B2 in near-neighbor species suggests that further screening will reveal additional species expressing *B. pseudomallei* O-antigen types.

In our present study, LPS genotyping and phenotypic analyses of numerous near-neighbor isolates suggested the presence of type A in *B. mallei*, *B. thailandensis*, and *B. oklahomensis*; type B in *B. ubonensis*; and type B2 in *B. thailandensis*, a *B. thailandensis*-like species, and *B. ubonensis*. Representative strains containing *B. pseudomallei* O-antigen ladder banding patterns were chosen for further whole genome sequencing and subjected to comparative genomics.

## Results

### 16S rRNA and *recA* sequencing

We confirmed bacterial species on all 113 bacterial strains using 16S rRNA and *recA* sequencing techniques compared to reference strains available in GenBank. Cutoffs of 99% and 94% were established for species classification for 16S and *recA* analyses, respectively (data not shown). We identified 23 *B. mallei*, 4 *B. oklahomensis*, 12 *B. thailandensis*, 5 *B. thailandensis*-like species, 44 *B. ubonensis*, and 25 unidentified *Burkholderia* species strains.

### LPS genotyping (PCR)

Eleven out of 12 *B. thailandensis* strains had the LPS genotype A. All 23 tested *B. mallei* strains also had the LPS genotype A. LPS genotype B was detected in 11 out of 44 strains of *B. ubonensis*. We note that these LPS genotype B strains were all of Australian origin. LPS genotype B2 was found in *B. thailandensis* strain 82172, and *B. thailandensis*-like species strains MSMB121, MSMB122, MSMB712, and MSMB714. This is the first reported incidence of another O-antigen in *B. thailandensis* while *B. thailandensis*-like MSMB121 was previously described as expressing this type [11]. No other species was positive for type A, B, or B2 (Table 1 and Additional file 1: Table S1).

**Table 1:** **Prevalence of four *B. pseudomallei *O-antigen types in near-neighbors**

**Species**	**Total strains tested**	**Known *B. pseudomallei *O-antigen**			
		**Type A**	**Type B**	**Type B2**	**Rough Type**
*B. mallei*	23	21	0	0	2
*B. oklahomensis*	4	1	0	0	0
*B. thailandensis*	12	11	0	1†	0
*B. thailandensis-like*	5	0	0	4	0
*B. cepacia*	2	0	0	0	0
*B. multivorans*	3	0	0	0	0
*B. ubonensis*	44	0	11	1‡	0
*B. vietnamiensis*	1	0	0	0	0
*Unidentified Burkholderia* spp.	19	0	0	1*	0

**†**Strain 82172, collected from French foal.

‡Strain MSMB108, collected from Northern Australian environment.

*Strain MSMB175, a soil strain collected from Australia. This strain is currently being proposed as a new *Burkholderia* species.

### LPS phenotyping (SDS-PAGE, silver staining and immunoblotting)

**Figure 1:**
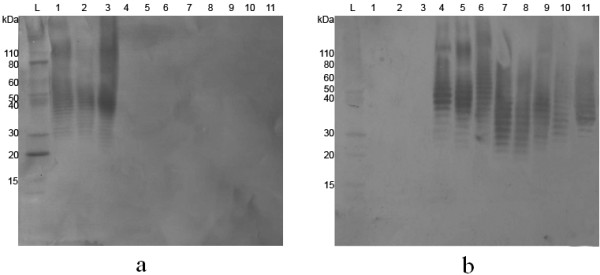
**Serotype A (a) and B (b) western blots.** Lane 1 – *B. pseudomallei* K96243, 2 – *B. thailandensis* E264, 3 – *B. oklahomensis* E0147, 4 – *B. pseudomallei* 576, 5 – *B. ubonensis* MSMB57, 6 – *B. pseudomallei* MSHR840, 7 – *B. thailandensis* 82172, 8 – *B. thailandensis*-like MSMB122, 9 – *B. ubonensis* MSMB108, 10 – *Burkholderia* sp. MSMB175, 11 – *B. thailandensis*-like MSMB43. Lanes 1–3 are representative of type A strains, Lanes 4–5 are representative of type B strains, Lanes 6–10 are representative of type B2 strains, and Lane 11 contains an unknown serotype B O-antigen

We identified LPS banding patterns in all tested bacterial strains by comparing them with known LPS banding patterns A, B, and B2 in reference *B. pseudomallei* strains (Additional file 2: Figure S1). Previously, only type A O-antigen has been described in *B. thailandensis*[11,12]. Eleven out of 12 tested strains expressed a type A banding pattern consistent with the PCR results. We note that *B. thailandensis* strain 82172 had the LPS genotype B2 via PCR, which was confirmed as serotype B by immunoblotting (Figure 1). *B. pseudomallei* strains expressing type B2 have previously been isolated only in Australia and Papua New Guinea, while this *B. thailandensis* strain was isolated in France [11,18]. Additionally, type A was recently described in *B. oklahomensis* E0147 [11], whereas the remaining three *B. oklahomensis* strains isolated from Oklahoma [19] displayed an unknown non-seroreactive ladder pattern (not shown in Figure 1).

Twenty-one strains of *B. mallei* expressed type A O-antigen while the remaining two strains (ATCC10399 and NCTC120) expressed rough type. ATCC10399 was previously described as having an intact ladder [13,20], but the whole genome sequence (WGS) available in GenBank shows an IS*407A* insertion in *wbiG* (NZ_CH899681), which would predict a rough type. IS*407A* is known as one of the most common insertion sequence (IS) elements in *B. pseudomallei* and *B. mallei*[21]. NCTC120’s rough type phenotype is consistent with prior works [13,20]. Further immunoblotting with the *B. mallei* LPS-specific mAb 3D11 showed all 21 *B. mallei* strains with intact ladder profiles bound this antibody while the two rough type strains did not. *B. pseudomallei* K96243 and *B. oklahomensis* E0147 bound mAb 3D11, as previously described [11]. Similarly, eight of the *B. thailandensis* strains bound mAb 3D11 while E264, MSMB59, MSMB60, and 82172 did not (Additional file 1: Table S1). Similarly, testing the strains containing type A with the IgM mAb Pp-PS-W, the *B. pseudomallei* LPS-specific mAb [13], showed that *B. mallei* ATCC23344 and *B. oklahomensis* E0147 were not seroreactive while *B. pseudomallei* K96243 was seroreactive. Notably, nine *B. thailandensis* strains were seroreactive to this mAb, while MSMB59 and MSMB60 were not. This suggested the existence of seroreactivity diversity within *B. thailandensis*.

PCR suggested that 11 strains of *B. ubonensis* would be positive for type B O-antigen. Immunoblotting confirmed the expression of type B in all of these, one of which, MSMB57, was selected for genomic analysis. Another strain, *B. ubonensis* MSMB108, was negative for all genotypes by PCR but displays a ladder pattern identical to the type B2 *B. thailandensis*-like MSMB122 (Figure 1). We also noted that other tested *B. ubonensis* strains produced distinct LPS ladder patterns to those of *B. pseudomallei*, which were not seroreactive (data not shown). Along with *B. thailandensis*, *B. ubonensis* was the only species that expressed more than one type of *B. pseudomallei* O-antigen.

*B. thailandensis*-like strains expressed two different O-antigen ladder patterns, both of which were B serotypes. Strains MSMB121, 122, 712, and 714 expressed ladder type B2 (Additional file 1: Table S1), whereas strain MSMB43 expressed a novel serologically related O-antigen not found in *B. pseudomallei*. This O-antigen, like type B2, bound the type B patient’s serum but exhibited a banding pattern unlike either type B or B2 (Figure 1). This is the first description of a seroreactive O-antigen found in a near-neighbor species which is unknown in *B. pseudomallei*.

*Burkholderia* sp. MSMB175 was negative for all *B. pseudomallei* O-antigen types by PCR. The immunoblotting analysis revealed a banding pattern that was similar to type B2 in higher molecular weight bands (Figure 1). The O-antigen biosynthesis gene cluster for this strain was subsequently sequenced and found to be type B2 (GenBank: JQ783347), with a nucleotide identity of 88% compared to *B. pseudomallei* MSHR840.

### Genomic analysis

Genomic comparison has shown that a homolog of *wbiE* gene in *B. oklahomensis* E0147 (BoklE_010100014785) had one and five single nucleotide polymorphisms (SNPs) at the forward and reverse primer binding sites, respectively. This caused negative PCR results when the previously published LPS genotype A primers [11] were used. In this study, we have adjusted the LPS genotype A primers to be able to amplify all *Burkholderia* species that contains the LPS genotype A. Similarly, in the type B2 positive *Burkholderia* sp. MSMB175, two and five SNPs were found in the forward and reverse primer pair binding sites, respectively, revealing why this strain was negative to PCR. In this study, we did not adjust the PCR primers to amplify the LPS genotype B2 in this uncharacterized *Burkholderia* species.

*B. thailandensis* E264, MSMB59, and MSMB60 were compared to determine the reason for the differences in sero-reactivity with the mAb Pp-PS-W. Four SNPs were found across the entire gene cluster, however all were synonymous and the amino acid sequences identical (data not shown). In addition, comparison of *oacA*, the 4-*O* acetyltransferase gene*,* sequences also revealed no differences. Further work is required to explain why the Australian isolates fail to cross react with this mAb.

Ten *Burkholderia* strains were selected for whole genome sequencing to confirm the LPS genotypes. These included *B. mallei* India 86-567-2, KC237, NCTC120; *B. thailandensis* MSMB59, MSMB60, 82172; *B. thailandensis*-like sp. MSMB121, MSMB122; *B. ubonensis* MSMB57; and *Burkholderia* sp. MSMB175. Comparative genomics has demonstrated that O-antigen biosynthesis genes in all three sequenced *B. mallei* strains were very similar to those found in a reference LPS genotype A *B. mallei* ATCC23344, except that strain NCTC120 had an insertion mutation in its *wbiE* gene (GenBank: JN581992). We noted that the mutation defects the production of O-antigen ladder pattern in this strain (Additional file 1: Table S1). In addition, genomic analysis has shown that O-antigen genes in *B. thailandensis* MSMB59 and MSMB60 were very similar to those found in a reference LPS genotype A *B. thailandensis* E264. Interestingly, *B. thailandensis* 82172, and *B. thailandensis*-like sp. strains MSMB121, MSMB122, and *Burkholderia sp*. MSMB175 had O-antigen genes similar to those found in a reference type B2 *B. pseudomallei* MSHR840, while *B. ubonensis* MSMB57 had O-antigen genes which were similar to the genes found in a reference type B *B. pseudomallei* 576 [11].

**Figure 2:**
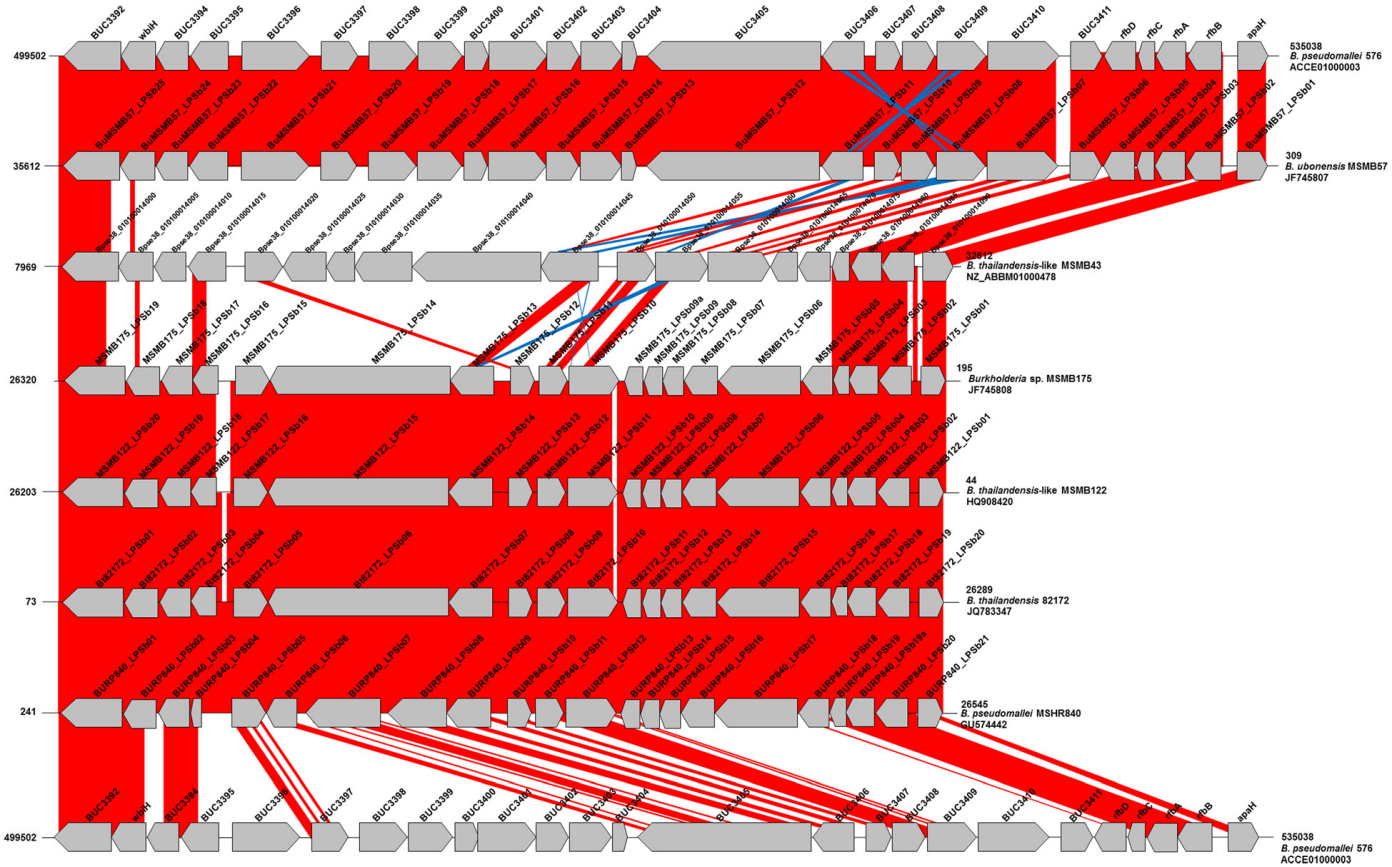
**Genomic comparison of O-antigen serotype B biosynthesis genes.** Gene clusters, from top to bottom, of *B. pseudomallei* 576 (type B), *B. ubonensis* MSMB57 (type B), *B. thailandensis*-like MSMB43 (type B variant), *Burkholderia* sp. MSMB175 (type B2), *B. thailandensis*-like MSMB122 (type B2), *B. thailandensis* 82172 (type B2), *B. pseudomallei* MSHR840 (type B2), and *B. pseudomallei* 576 were used to illustrate the diversity of the serotype B O-antigen biosynthesis gene clusters. Red indicates homology of 78-100% and blue indicates an inversion region with equal homology

One strain of each species expressing O-antigen types A, B, or B2 were selected for further genomic comparisons. The type A strains *B. pseudomallei* K96243, *B. mallei* ATCC23344, *B. thailandensis* E264, and *B. oklahomensis* E0147 had an overall nucleotide similarity of 87.2% to each other, a genic similarity of 87.2%, and an amino acid similarity of 88.7% (Additional file 3: Figure S2). The type B strains *B. pseudomallei* 576 and *B. ubonensis* MSMB57 had an overall nucleotide similarity of 95%, a genic similarity of 95%, and an amino acid similarity of 95%. The type B2 strains *B. pseudomallei* MSHR840, *B. thailandensis* 82172, *B. thailandensis*-like MSMB122, and *Burkholderia* sp. MSMB175 had an overall nucleotide similarity of 90.2%, a genic similarity of 88%, and an amino acid similarity of 86.5%. The diversity of genes that are predicted to be involved in the biosynthesis of LPS types B and B2 is demonstrated in Figure 2. Comparison of the novel B serotype found in *B. thailandensis*-like MSMB43 with types B and B2 revealed a conservation of the putative epimerase *wbiI* and rhamnose synthesis genes *rmlCAB* (Figure 2) [11,22]. Transport genes, e.g., ABC-transporters, encoding two *wzt* and one *wzm* homologs, are conserved across all three serotype B ladder types. These *wzt* and *wzm* homologs are genes BUC_3406, BUC_3409, BURP840_LPSb09, BURP840_LPS12, Bpse38_010100014045, Bpse38_010100014055, and genes BUC_3408, BURP840_LPSb11, Bpse38_010100014050, respectively (Figure 2). These gene products are likely responsible for the sero-crossreactivity observed between these O-antigens (Figure 1). However, a glycosyl transferase gene, Bpse_38010100014060 in *B. thailandensis*-like MSMB43, which is similar to those found in type B ladder (gene BUC_3410 in *B. pseudomallei* 576 and gene BuMSMB57_LPSb07 in *B. ubonensis* MSMB57) has no homology to any of those in the type B2. The type A strains displayed the greatest level of nucleotide diversity, suggesting an ancient acquisition of the gene cluster and a possible ancestral state. Conversely, the type B strains were the most monomorphic, albeit with fewer species representative of this type. In addition, the average G+C content of each cluster was 60.8% for type A, 61% for type B, and 63.5% for type B2. Given an average genomic G+C content of 68.1% for the Pseudomallei group, the observed G+C content of the O-antigen gene clusters is evidence for horizontal acquisition. This would suggest, however, that type A was unlikely the ancestral type despite being the most abundant and genetically diverse today.

### Serum sensitivity

**Figure 3:**
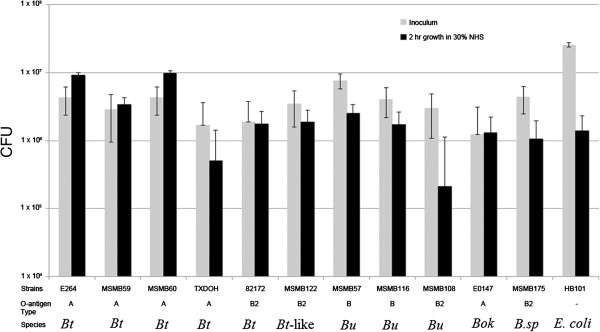
**Serum sensitivity of *B. pseudomallei *near-neighbors.***B. thailandensis* E264, MSMB59, MSMB60 and *B. oklahomensis* E0147 showed a slight resistance to killing by 30% NHS while all other strains were susceptible to killing, especially *B. ubonensis* MSMB108. This is in agreement with prior studies showing serum sensitivity of *B. pseudomallei* strains expressing type B2 or rough type O-antigens. Note: *Bt*, *B. thailandensis*; *Bt*-like, *B. thailandensis*-like species; *Bu*, *B. ubonensis*; *Bok*, *B. oklahomensis*; and *B.sp*, *Burkholderia* sp

Previous studies have shown that *B. pseudomallei* strains with type B2 or rough type O-antigens display an increased sensitivity to killing by 30% NHS [11,23]. To determine if near-neighbors showed the same effect, eleven diverse *Burkholderia* strains expressing type A, B, or B2 O-antigen were assayed for serum sensitivity. All type A strains, *B. thailandensis* E264, MSMB59, MSMB60, and *B. oklahomensis* E0147 showed a slight resistance to serum killing, except *B. thailandensis* TXDOH which was sensitive to serum killing. The type B2 *B. thailandensis* 82172 showed almost no difference in growth, and all other strains were sensitive to killing by 30% NHS, most notably *B. ubonensis* MSMB108 (Figure 3).

## Discussion

O-antigen type A has been described as a disaccharide glucose-talose repeat in *B. pseudomallei*, *B. mallei*, and *B. thailandensis* and these structures differ only by side group modification. *B. pseudomallei* modifies the talose residue with a 2-*O* methyl/4-*O* acetyl group or with a 2-O acetyl/4-*O* hydroxyl group [15,16]. In *B. mallei*, regardless of whether the 2-*O* position is methylated or acetylated, the 4-*O* position remains in its native hydroxyl state [13]. *B. thailandensis* has been reported to have the same modification patterns as *B. pseudomallei*[12,14,22], but a recent study by Ngugi, *et al.,*[10] suggests that *B. thailandensis* E264 features a different pattern. Utilizing gas chromatography/mass spectrometry (GC/MS) to examine methylation patterns, they concluded this strain does not methylate the 2*-O* position. Brett, *et al.,*[14] generated mutants of *oacA,* the 4*-O* acetyltransferase gene, which also had the unexpected result of a lack of methylation at the 2-*O* position. This suggests that the methyl group may be lost during GC/MS or the E264 strain utilized by Ngugi, *et al.,*[10] may have undergone mutation in *oacA*, losing its methylase capabilities.

In our current study, 21 out of 23 *B. mallei* strains expressed intact type A O-antigens while the remaining two (ATCC10399 and NCTC120) were rough. Two previous studies showed that *B. mallei* ATCC10399 had a full ladder pattern by silver staining and immunoblotting [13,20]. Our genomic analysis has shown that *wbiG* gene which is known to be involved in the biosynthesis of the type A O-antigen, was disrupted in this strain by IS*407A*. This supported our finding that ATCC10399 produced rough LPS. *B. mallei* NCTC120 was also known as a rough LPS type due to the disruption of its *wbiE,* the glycosyltransferase gene, by IS*407A*[13,20]. DNA sequencing of this strain in our current study revealed the absence of this insertion element, however, a 22 base pair artifact remains in the 3^′^ end of this gene (GenBank: JN581992), suggesting, IS*407A* remains active in this strain. We believe that the artifact sequence of the IS*407*A is disruptive enough to yield the same phenotype as the full insertion.

Eleven strains of *B. ubonensis*, all Australian environmental isolates, were found to express type B. This O-antigen type is present in approximately 14% of all *B. pseudomallei* isolates of which the vast majority are Australian [11]. We report here the first discovery of *B. pseudomallei* type B O-antigen in a near-neighbor species. Previously, *B. ubonenesis* was known in Australia from only two strains, only one of which has been sequenced and contains an unknown O-antigen biosynthesis gene cluster (NZ_ABBE01000374) [24]. Environmental sampling in northern Australia yielded 44 total *B. ubonensis* strains, which was the species most commonly isolated. Conversely, only two *B. thailandensis* strains were isolated, the same number as Levy, *et al.,* found [24]. While no study has examined the abundance of *B. ubonensis* in Southeast Asia, it is possible that these two species occupy a similar environmental niche where *B. ubonensis* is able to outcompete *B. thailandensis* in Australia. In support this, *B. ubonensis* isolated from Papua New Guinea exhibited antibiosis against *B. pseudomallei*[25]. These Australian isolates may produce a similar compound against *B. thailandensis*.

*B. thailandensis*-like species, a new member of the Pseudomallei group, expresses type B2 and a novel ladder pattern seropositive for type B, thus far unknown in any other species or strain. Curiously, *B. thailandensis* 82172 expresses type B2, as well, marking the first description of another O-antigen type in this species. This strain belongs to a distinct phylogenetic cluster along with four other geographically diverse *B. thailandensis* strains, only one of which was isolated in Asia. This cluster has been suggested as the beginning of a possible speciation event and the discovery of type B2 LPS lends further credence to this idea [26].

*Burkholderia* sp. MSMB175 is another Australian environmental isolate which clusters with the Pseudomallei group on the basis of *recA* and 16S sequence and may represent a new species (data not shown). The presence of type B2 O-antigen (Table 1) supports the possibility that this strain belongs to the Pseudomallei group.

A 1993 study of northeastern Thai children by Kanaphun, *et al.,*[27] revealed that 80% are seropositive for antibodies against *B. pseudomallei* by the age of four. Accordingly, over 25% of environmental *Burkholderia* isolates in Thailand are *B. thailandensis*[28]. This suggests that some of these children have instead been exposed to this species and not *B. pseudomallei*, especially given the noted inaccuracies and high background of indirect hemagglutination assays [29]. Little work has examined the seropositive rates in Australia, but two studies in Northern Queensland returned rates of 2.5-5.7% [30,31]. The high clinical relevance of *B. pseudomallei* expressing type B or B2 O-antigen, along with the new apparent abundance of these types in Australian near-neighbors, suggest similar exposures may result in false positive diagnoses, as is likely the case in Thailand.

These near-neighbor species are avirulent, *B. mallei* excepted, and as such are not limited to the biosafety regulations that *B. pseudomallei* is as a biosafety level 3 (BSL-3) organism. Few laboratories worldwide are properly equipped to handle BSL-3 work and so the finding of *B. pseudomallei* type LPS in these non-pathogenic *Burkholderia* species will allow many additional laboratories the opportunity to work towards vaccine development for melioidosis.

## Conclusions

*B. thailandensis* type A O-antigen has been used with some success to vaccinate mice against *B. pseudomallei*[7-10]. This O-antigen is indistinguishable between these two species in backbone and side group modifications [12,16,22]. Given the high genetic similarity between types B and B2 in near-neighbors and *B. pseudomallei*, it is likely at least one species will be identical in backbone and side group modifications, as well. In such a case, it is possible that particular strain or strains will confer comparable host immunity upon subsequent challenge with type B or B2 *B. pseudomallei* in much the same way *B. thailandensis* protects against type A *B. pseudomallei* challenge.

## Methods

### Bacterial strains, DNA, and LPS preparations

A total of 113 strains of *B. pseudomallei* near-neighbors were used in this study. These included 23 *B. mallei*, 4 *B. oklahomensis*, 12 *B. thailandensis*, 5 *B. thailandensis*-like species, 44 *B. ubonensis*, and other 25 *Burkholderia* strains (Tables 1 and Additional file 1: Table S1). Species identification was made on the basis of *recA* and 16S rRNA sequences [17,18]. *B. pseudomallei* strains K96243, 576, MSHR840, and MSHR1655 were used as references for the O-antigen types A, B, B2, and rough, respectively [11]. All strains were grown on Luria-Bertani (LB) agar (Difco, USA) for DNA and LPS extractions. DNA was extracted using the Wizard Genomic DNA Purification Kit (Promega, Madison, WI, USA), according to the manufacturer’s instructions. LPS was extracted using whole-cell lysis according to a previous method [11,20] and separated by SDS-PAGE (Invitrogen, USA).

### PCR analysis

Strains were genotyped for *B. pseudomallei* O-antigen types via multiplex-SYBR-Green real-time PCR in accordance with as previously reported [11]. As the previously published sequences did not detect all near-neighbors expressing type A, this primer pair was redesigned. The new type A primer pair spans the intergenic space of *wbiD* and *wbiE* and the primer sequences are: LPSA_For, 5^′^-ACGGGATCGTACAGTTTCGGATGCT-3^′^; and LPSA_ Rev, 5^′^-GAAGATCGTCGCTCGGAGAATCGT-3^′^.

### Silver staining and serology

Silver staining was first used to validate *B. pseudomallei* O-antigen type presence in near-neighbor strains, following the previously determined criteria for identification [11,20]. Samples were then screened for sero-crossreactivity using sera from two Australian melioidosis patients, one serum sample per immunoblot analysis. One patient was infected by *B. pseudomallei* MSHR1328 expressing type A O-antigen, while another patient was infected by strain MSHR1079 which expressed type B O-antigen [11]. The same samples were also tested serologically using the commercially available monoclonal antibody (mAb) 3D11 (Fitzgerald Industries International Inc., USA), specific to *B. mallei* LPS [23]. Additionally, LPS samples from all *B. thailandensis* strains were also tested using mAb Pp-PS-W [13] which is specific to *B. pseudomallei* type A O-polysaccharide (O-PS).

### Serum-sensitivity testing

The susceptibility of the near-neighbor strains to 30% normal human serum (NHS; Lonza Group LtD., USA) was tested according to a previous method [11,23]. Briefly, strains were grown at 37°C overnight with shaking in LB broth and cell concentrations were equilibrated. A 1:1,000 dilution of culture was created in TSB-DC (Trypticase soy broth dialysate –treated with Chelex-100) media [32], and grown for five hours. A 1:6:3 vol. ratio of the culture: TSB-DC media:undiluted NHS was incubated for two hours at 37°C with no shaking. Total bacterial plate counting was performed on these cultures. *E. coli* HB101 was used as a negative control.

### Whole genome sequencing and genomic analysis

Whole genome sequencing was performed using 454 sequencing technology (Roche, USA) by the US Army Edgewood Chemical Biological Center (ECBC), Aberdeen, MD. O-antigen biosynthesis gene cluster annotations were made in comparison to the aforementioned reference *B. pseudomallei* types using the BLAST program and Artemis Comparison Tool (ACT) [33]. Annotated O-antigen gene sequences of *B. mallei* strains India 86-567-2, KC237, NCTC120; *B. thailandensis* strains MSMB59, MSMB60, 82172; *B. thailandensis*-like species strains MSMB121, MSMB12; *B. ubonensis* strain MSMB57; and unidentified *Burkholderia* sp. strain MSMB175, were assigned GenBank accessions: JN581990, JN581991, JN581992, JN581997, JN581998, JQ783347, HQ908420, JF745809, JF745807, and JF745808, respectively.

## Competing interests

Authors declare that they have no competing interests.

## Authors’ contributions

AT, BJC and PK conceived of the study. JKS performed major experimental analyses and drafted the manuscript. MM, SAG, JLG, CJA, AD, SG, and MK provided technical assistances. HSG, SMB, MAK, JMI, KSH, and LAM sequenced all *Burkholderia* genomes used in this study. PK, BJC, and AT reviewed and edited the manuscript. All authors read and approved the final manuscript.

## Supplementary Material

Additional file 1 **Table S1.** List of *Burkholderia* strains used in this study, and their identified genotypes and phenotypes. Click here for file

Additional file 2 **Figure S1.** SDS-PAGE and immunoblotting analyses of 3 reference LPS banding patterns A, B, and B2 in *B. pseudomallei* strains K96243 (lane 1), 576 (lane 2), and MSHR840 (lane 3), respectively. Panel A is the silver stained SDS-PAGE. Panels B and C are the immunoblots of LPS samples in panel A which were hybridized against sera from serotype A and B patients, respectively. Lane 4 is the LPS from *B. pseudomallei* strain MSHR1655 which is rough type and not seroreactive. Lane L is a standard protein ladder. Click here for file

Additional file 3 **Figure S2. **Comparison of type A O-antigen biosynthesis clusters. Type A O-antigen is found in four species, from top to bottom, *B. oklahomensis*, *B. pseudomallei*, *B. mallei*, and *B. thailandensis*. Red indicates nucleotide homology of 78-100%. The glycosyltransferase gene *wbiE* (BoklE_010100014785) is truncated in *B. oklahomensis* E0147 but maintains functional. Conversely, insertion of a thymine into the methyltransferase *wbiD* relative to *B. pseudomallei* K96243 removes the functionality of this enzyme in E0147, removing it from the comparison. Click here for file
