# Supplementary figures and images for: Detection of Burkholderia pseudomallei O-antigen serotypes in near-neighbor species
Source: BMC Microbiol. 2012 Nov 5;12:250. doi: 10.1186/1471-2180-12-250 (PMC3541218; doi:10.1186/1471-2180-12-250)

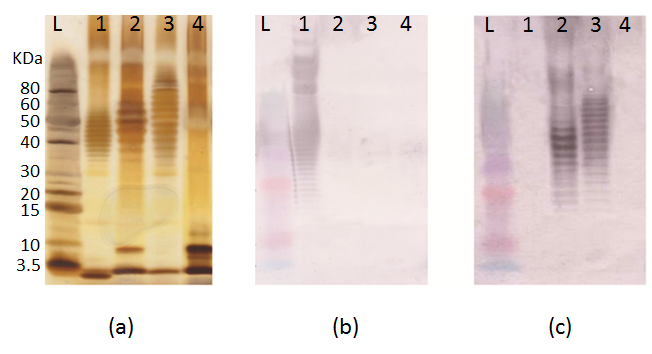

Supplement: Additional file 2 — Figure S1. SDS-PAGE and immunoblotting analyses of 3 reference LPS banding patterns A, B, and B2 in B. pseudomallei strains K96243 (lane 1), 576 (lane 2), and MSHR840 (lane 3), respectively. Panel A is the silver stained SDS-PAGE. Panels B and C are the immunoblots of LPS samples in panel A which were hybridized against sera from serotype A and B patients, respectively. Lane 4 is the LPS from B. pseudomallei strain MSHR1655 which is rough type and not seroreactive. Lane L is a standard protein ladder. [file 1471-2180-12-250-S2.png]

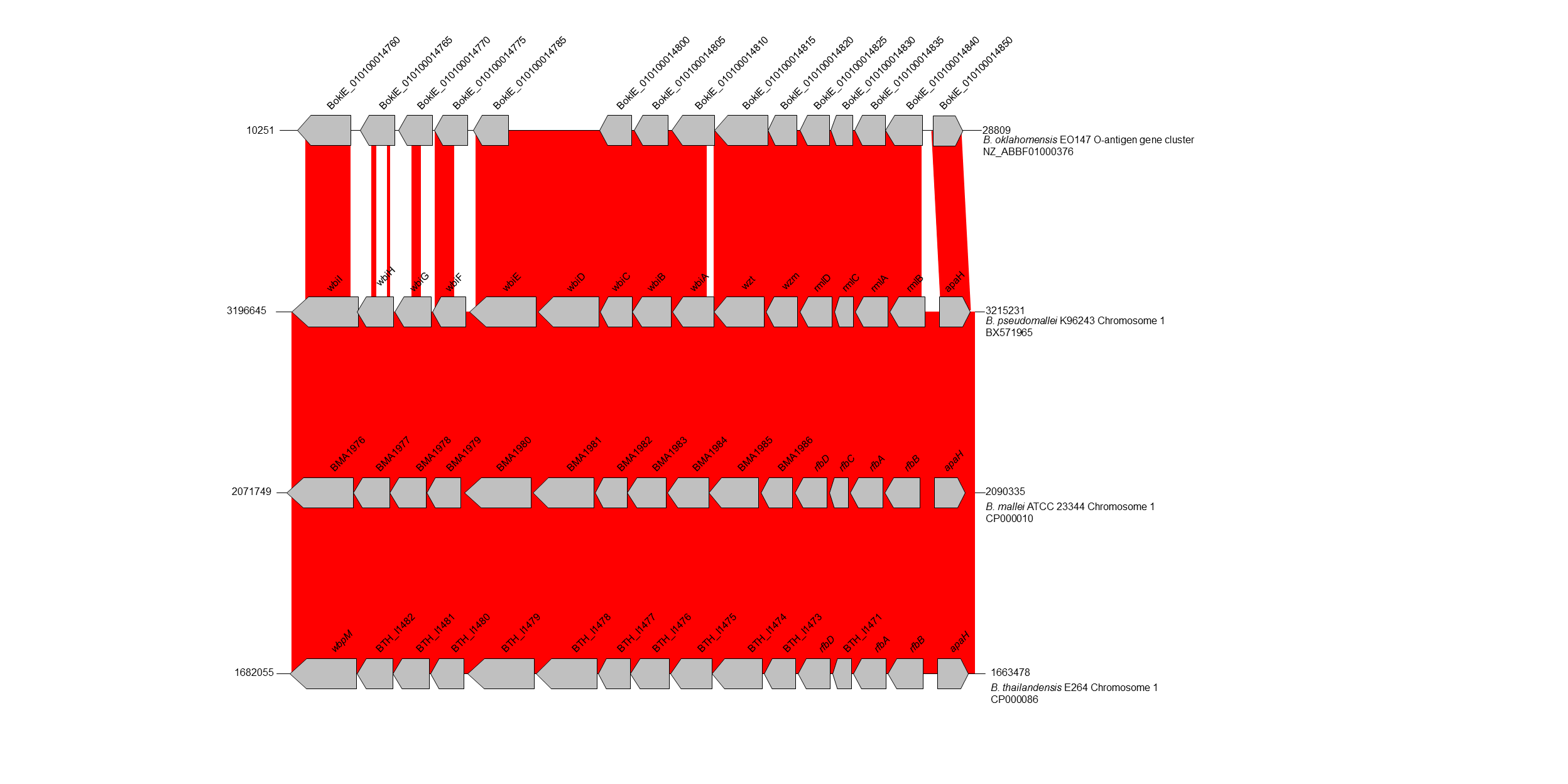

Supplement: Additional file 3 — Figure S2. Comparison of type A O-antigen biosynthesis clusters. Type A O-antigen is found in four species, from top to bottom, B. oklahomensis, B. pseudomallei, B. mallei, and B. thailandensis. Red indicates nucleotide homology of 78-100%. The glycosyltransferase gene wbiE (BoklE_010100014785) is truncated in B. oklahomensis E0147 but maintains functional. Conversely, insertion of a thymine into the methyltransferase wbiD relative to B. pseudomallei K96243 removes the functionality of this enzyme in E0147, removing it from the comparison. [file 1471-2180-12-250-S3.png]
